# Immunization with plasmid DNA expressing Heat Shock Protein 40 confers prophylactic protection against chronic *Toxoplasma gondii* infection in Kunming mice

**DOI:** 10.1051/parasite/2018040

**Published:** 2018-07-23

**Authors:** Zhong-Yuan Li, Jing Lu, Nian-Zhang Zhang, Hany M. Elsheikha, Jun-Ling Hou, Hai-Ting Guo, Xing-Quan Zhu

**Affiliations:** 1 College of Animal Science and Technology, Anhui Agricultural University Hefei Anhui 230036 PR China; 2 State Key Laboratory of Veterinary Etiological Biology, Key Laboratory of Veterinary Parasitology of Gansu Province, Lanzhou Veterinary Research Institute, Chinese Academy of Agricultural Sciences Lanzhou Gansu 730046 PR China; 3 Faculty of Medicine and Health Sciences, School of Veterinary Medicine and Science, University of Nottingham, Sutton Bonington Campus Loughborough LE12 5RD UK; 4 College of Biological Science and Technology, Heilongjiang Bayi Agricultural University Daqing Heilongjiang 163319 PR China

**Keywords:** *Toxoplasma gondii*, HSP40, DNA vaccine, Chronic infection, Immune evaluation

## Abstract

*Toxoplasma gondii* causes one of the most common protozoal diseases of humans and animals worldwide. With the aim of designing an effective vaccine against *T. gondii* infection, we examined the immunogenicity of a DNA vaccine expressing heat shock protein 40 (HSP40) against challenge with *T. gondii* (type I RH and type II Pru) strains in Kunming mice. The plasmid pVAX1-HSP40 was constructed and used to immunize mice by intramuscular injection for three sequential immunizations with two-week intervals. This immunization regimen significantly reduced parasite cyst burden in pVAX1-HSP40-immunized mice (1871.9 ± 142.3) compared with control mouse groups immunized with pVAX1 (3479.2 ± 204.4), phosphate buffered saline (3024.4 ± 212.8), or left untreated (3275.0 ± 179.8) as healthy controls (*p* < 0.01). However, immunization failed to protect mice against challenge with the virulent RH strain. There was a significant increase in T lymphocyte subclasses (CD3e^+^CD4^+^ T and CD3e^+^CD8a^+^ T lymphocytes) in splenic tissues in immunized mice compared with controls (*p* < 0.05). However, the level of antibodies, lymphocyte proliferation and concentration of cytokines (IFN-*γ*, IL-2, IL-4, IL-10 and IL-12p70) were not significantly different between immunized and control mouse groups (*p* < 0.05). These data indicate that pVAX1-HSP40 induced specific immune responses and achieved a significant reduction in the number of brain cysts in Pru-infected mice, and thus can be tested in future immunization studies along with plasmids containing other immunogenic proteins as a cocktail vaccine to fully abolish chronic toxoplasmosis.

## Abstract (fr)

*Toxoplasma gondii* provoque l’une des maladies à protozoaires les plus courantes chez les humains et les animaux dans le monde entier. Dans le but de concevoir un vaccin efficace contre l’infection à *T. gondii*, nous avons examiné l’immunogénicité d’un vaccin ADN exprimant la protéine de choc thermique HSP40 contre des souches de *T. gondii* (type I RH et type II Pru) chez des souris Kunming. Le plasmide pVAX1-HSP40 a été construit et utilisé pour immuniser des souris par injection intramusculaire pour trois immunisations séquentielles avec des intervalles de deux semaines. Ce schéma d’immunisation a réduit significativement la charge de kyste parasitaire chez les souris immunisées par pVAX1-HSP40 (1871.9 ± 142.3) par rapport aux groupes de souris témoins immunisés avec pVAX1 (3479.2 ± 204.4), traités par solution saline tamponnée au phosphate (3024.4 ± 212.8) ou non traités (3275.0 ± 179.8) comme témoins sains (*p* < 0.01). Cependant, l’immunisation n’a pas réussi à protéger les souris contre la souche virulente RH. Il y avait une augmentation significative des sous-classes de lymphocytes T (lymphocytes T CD3e^+^ CD4^+^ T et CD3e^+^CD8a^+^) dans les tissus spléniques chez les souris immunisées par rapport aux témoins (*p* < 0.05). Cependant, le taux d’anticorps, la prolifération lymphocytaire et la concentration de cytokines (IFN-γ, IL-2, IL-4, IL-10 et IL-12p70) n’étaient pas significativement différents entre les groupes de souris immunisés et témoins (*p* < 0.05). Ces données indiquent que pVAX1-HSP40 induit des réponses immunitaires spécifiques et réduit significativement le nombre de kystes cérébraux chez les souris infectées par Pru, et peut donc être testé dans des études de vaccination futures avec des plasmides contenant d’autres protéines immunogènes dans un cocktail de vaccins, pour abolir la toxoplasmose chronique.

## Introduction

The Apicomplexan protozoan *Toxoplasma gondii* is the agent of a major zoonotic disease, toxoplasmosis, affecting mainly immunocompromized individuals and naïve pregnant women [3, 5, 8, 29]. Current therapeutic medicines are not highly effective or cause adverse effects [1, 36]. Currently, there are no approved human vaccines against toxoplasmosis disease, and efforts are ongoing to identify protective antigens and the best strategies for vaccine development [17, 35, 39]. Effective therapeutic interventions are thus desirable and will be facilitated by a better understanding of the extent of the efficacy achieved by vaccination using key proteins to prevent *T. gondii* infection.

Heat shock proteins (HSPs) are ubiquitous constitutively or inducibly expressed proteins that act as molecular chaperones assisting in the assembly, folding, stabilization, and translocation of other cellular proteins. They are generally upregulated in response to various stress conditions [33], and bind unfolded, misfolded, or denatured proteins to prevent unwanted aggregation [32]. HSPs play roles in cell cycle progression, and transcriptional and posttranslational processes, such as protein folding, stability, transportation, and degradation, and they also play roles in the pathogenesis of inflammation and cancer [20, 27, 37]. HSPs are highly effective and versatile molecules in promoting immune responses against tumors and infections [7]. They can also mediate antigen presentation and activation of immune cells, such as lymphocytes, macrophages and dendritic cells [6]. They are presumed to have immunogenic properties due to their ability to bind, stabilize, and protect the antigen from degradation [34].

HSP40 plays a regulatory role in DNA duplication, protein modification, degradation and translocation across the membrane, endocytosis, and cell-signal transduction [15, 24]. It is involved in the pathogenicity of viruses such as *Simian virus 40* [21] and protozoa such as *Plasmodium falciparum* [30]. HSP40 plays an essential role in the mechanisms of *T. gondii* bradyzoite development [9], and the nucleotide sequences of HSP40 genes are highly conserved among *T. gondii* genotypes, indicating that HSP40 might be a good vaccine candidate to counter the development and dissemination of *T. gondii* [23]. In this study, we tested the hypothesis that a DNA vaccine could provide a safe and reliable strategy against acute and chronic infection with *T. gondii*, and that HSP40 is a target of protective immune responses. Our results indicate that HSP40-based DNA vaccine effectively inhibited parasite cyst burden and induced antigen-specific adaptive cellular immune response.

## Materials and methods

### Ethics statement

All animal experiments were performed strictly in accordance with the guidelines of the Animal Ethics Procedures of the People’s Republic of China. Ethical approval to conduct the procedures that involved mouse immunization and parasite challenge was obtained from the Animal Ethics and Administration Committee of Lanzhou Veterinary Research Institute, Chinese Academy of Agricultural Sciences (Approval No. LVRIAEC2012-011).

### Animals and parasites

Female, 6–8-week old specific pathogen-free (SPF) grade, Kunming mice were purchased from the Laboratory Animal Center of Lanzhou Biological Product Institute (Lanzhou, China). Mice were housed in a High-density Touch Screen Mouse IVC System (FENGSHI, Qingdao, China), and their food and bedding materials were provided by Beijing Keao corporation (http://u6452366.b2bname.com/). Tachyzoites of *T. gondii* RH strain (Genotype I) maintained *in vitro* in African green monkey kidney cells and cysts of *T. gondii* Pru strain (Genotype II) separated from brain tissues of orally Pru-infected Kunming mice were used in the parasite challenge experiments to test the efficacy of the immunization. *T. gondii* lysate antigen (TLA) was prepared as described previously [22].

### Constructions of recombinant pET30a-HSP40 and pVAX1-HSP40 plasmids

Total RNA was extracted from *T. gondii* RH tachyzoites using the E.Z.N.A.^®^ Total RNA Kit I (Omega, Norcross, Georgia, USA). Based on the reference sequence of the ME49 strain (ToxoDB: TGME49_265310), a pair of specific primers (forward primer: 5′-GGGGTACCATGGGGAAGGACTACTACAGAA-3′; reverse primer: 5′-CGCGGATCCCTACACGTTCGGAAGCAGTT-3′) was designed and used to amplify the coding sequence of the *T. gondii* HSP40 gene. The *Kpn*I and *Bam*HI restriction sites are underlined in the primers. RT-PCR was carried out following the instructions of the PrimeScript^®^ One Step RT-PCR Kit (TaKaRa, Dalian, China). The amplification reaction was performed in the thermocycler with the following program: a reverse transcription reaction at 50 °C for 0.5 h; then denaturation at 94 °C for 2 min; followed by 35 cycles of denaturation at 94 °C for 1 min, annealing at 56.6 °C for 30 s, extension at 72 °C for 1.5 min, and was ended with a final extension at 72.0 °C for 10 min. The PCR products were purified using the E.Z.N.A.^®^ Gel Extraction Kit (Omega) and were inserted into the pMD18-T linear vector (TaKaRa), generating plasmid pMD-HSP40. The pMD-HSP40 plasmids were digested using the restriction enzymes *Kpn*I/*Bam*HI in order to obtain the HSP40 fragment. Then, the pET30a(+) or pVAX1 plasmids were digested using the same restriction enzymes, and the HSP40 fragment was ligated to them using T4 DNA ligase (TaKaRa), constructing the recombinant pET30a-HSP40 or pVAX1-HSP40 plasmids, respectively. The sequences of the inserts were confirmed by sequencing. The pET30a-HSP40 plasmid was used to prepare recombinant HSP40 protein (rHSP40). The concentration of pVAX1-HSP40 was measured at 260 nm and 280 nm using a BioMate 3S spectrophotometer (Thermo, Waltham, Massachusetts, USA). The plasmid was adjusted to a final concentration of 1 μg/μL.

### Preparation of rHSP40 protein

The pET30a-HSP40 plasmid was constructed using the same method and primers used to construct the pVAX1-HSP40 plasmid above. This pET30a-HSP40 plasmid was transfected into *Escherichia coli* BL21 competent cells by heat shock at 42 °C for 1.5 min, and was induced to express the rHSP40 protein *in vitro* using isopropyl-*β*-D-thiogalactoside (IPTG, Promega, Fitchburg, Wisconsin, USA). The products with 6-His tag were purified by Ni-NTA His Bind^®^ Resin (Novagen, Madison, Wisconsin, USA). The purified rHSP40 protein was used to coat the wells of the 96-well plates in the enzyme-linked immunosorbent assay (ELISA) to measure the concentrations of specific anti-rHSP40 antibodies.

### Transfection and expression of pVAX1-HSP40 *in vitro*

To confirm whether the HSP40 protein can be expressed in eukaryotic cells, we used human embryonic kidney cells 293 (HEK293), indirect immunofluorescence and Western blotting analyses, as described previously [13]. HEK293 cells were seeded at a concentration of 2.5 × 10^5^ cells/well into a six-well tissue culture plate until the cells reached ≥70% confluence. Transfection with pVAX1-HSP40 or empty pVAX1 (vector control) was performed with LipofectAMINE 2000 reagent (Invitrogen, Carlsbad, California, USA), as instructed by the manufacturer. Approximately 72 h post-transfection, the cells were fixed with 4% paraformaldehyde (PFA) for 30 min, permeabilized with 0.1% Triton X-100 for 30 min, and incubated with goat anti-*T. gondii* serum (1:50 in PBS) and the Alexa Fluor^®^ 488-AffiniPure donkey anti-goat IgG (H + L) diluted 1:1000 in PBS (Jackson ImmunoResearch Inc., West Grove, Pennsylvania, USA) was added and the samples were kept at ambient temperature in the dark for 60 min. The fluorescent images were obtained using a Zeiss Axioplan 2 fluorescence microscope (Carl Zeiss, Oberkochen, Germany).

Next, ~10^7^ cells (transfected with either pVAX1-HSP40 or pVAX1) were collected at 72 h post transfection, suspended into 200 μL of SDS-PAGE loading buffer (Sangon, Shanghai, China) and incubated at 100 °C for 10 min. The products were separated using 5%–12% gradient bis-tris gels (Sangon) and transferred to the nitrocellulose membrane (PALL, Port Washington, New York, USA) using a TRANS-BLOT^®^ SD CELL (Bio-Rad, Hercules, California, USA). The membrane was blocked with 5% non-fat milk in PBS and incubated with goat anti-*T. gondii* serum (1:50 in PBS), and subsequently with HRP-conjugated rabbit anti-goat IgG (H + L) (Agrisera, Vännäs, Sweden) (1:5000 in PBS). A DAB-HRP Color Development Kit was used, following the manufacturer’s instructions (CWBIO, Beijing, China).

### Immunization protocol

Kunming mice were randomly allocated into four groups (42 mice per group) and intramuscularly (i.m.) immunized three times at a two-week interval. The experimental mouse groups included: Group I injected with 100 μL of sterile phosphate-buffered saline (PBS; pH 7.4) containing 100 μg pVAX1-HSP40; Group II injected with 100 μL of PBS containing 100 μg of the empty pVAX1 (vector control); Group III injected with 100 μL of sterile PBS alone; and Group IV received no treatment and served as a healthy control. Spleen tissues collected aseptically from immunized and control mice (*n* = 3 mice per group) prior to the challenge were chopped with scissors into small pieces, and the splenic tissue fragments were pressed gently through a fine nylon mesh in order to prepare single cell suspensions.

### Efficacy of immunization against acute and chronic toxoplasmosis

The aim of these experiments was to test the efficacy of immunization with pVAX1-HSP40 against acute and chronic infections in mice. First, we tested the efficacy against acute infection two weeks after the final/third immunization by intraperitoneal inoculation (*n* = 15 mice/group) with 10^3^ tachyzoites of a virulent *T. gondii* RH strain. The survival times of all challenged mice were recorded daily. Secondly, we evaluated the level of protection induced by pVAX1-HSP40 against chronic infection in mice (*n* = 15/group) challenged with 10 cysts of a mildly virulent cyst-forming Pru strain of *T. gondii*. Mice were euthanized at 30 days post-challenge and their brains were removed and individually homogenized in 1 mL of PBS. The parasite cyst burden in the brain tissues of the mice was determined by examining dilutions of DBL-stained brain homogenates using a Zeiss wide-field epifluorescence microscope with 10× objective, as previously described [12].

### Determination of T lymphocytes in spleen tissue

Splenic single-cell suspensions prepared from all mice prior to the challenge were used to analyze T lymphocytes using flow cytometry, as previously described [22]. Briefly, 10^5^ viable cells counted using a hemocytometer and 0.04% trypan blue dye (Bio-Rad) were suspended in 100 μL of 2% FBS in PBS solution and incubated with 0.45 μL of FITC anti-mouse CD3e, 0.25 μL of APC-eFluor780 anti-mouse CD4 or 0.25 μL of Percp-cy5.5 anti-mouse CD8a (eBioscience, San Diego, California, USA) at 4 °C for 30 min in the dark. After two washes with 2 mL of PBS, the samples were re-suspended in 300 μL of FACScan buffer (0.1% NaN_3_, 1% BSA) and 250 μL of 2% PFA solution for optical measurement. The data from three independent experiments were obtained using the FACScan flow cytometer and SYSTEM II software (BD Bio-sciences, San Jose, California, USA).

### Antibody detection using ELISA

Blood samples were collected from the ophthalmic veins of three mice per group to assess the level of specific anti-anti-rHSP40 antibodies prior to each of the three immunization doses and prior to the challenge using ELISA. Flat-bottom 96-well plates were coated overnight at 4 °C with 100 μL of rHSP40 protein containing approximately 1 μg of the protein per well. Test sera diluted at 1:10 were added to the wells and incubated for 1 h at 37 °C. The antibody measurement was carried out using SBA Clonotyping^TM^ System/HRP Kit (SouthernBiotech, Birmingham, Alabama, USA), according to the manufacturer’s instructions (https://www.southernbiotech.com/techbul/5300-05.pdf). The optical density was measured using an ELISA reader (Bio-TekELx800, Winooski, Vermont, USA) at 405 nm (OD_405_). Each sample was tested in triplicate.

### Lymphocyte proliferation assay

Splenic lymphocytes isolated from mice prior to challenge were cultured in 96-well plates at a concentration of 3 × 10^4^ cells/well in 100 μL RPMI 1640 medium containing 10% fetal bovine serum in the presence of 10 μg/mL of ultrasonic extracts of whole *T. gondii* tachyzoites (TLA) for 72 h at 37 °C in a 5% CO_2_ incubator. Lymphocytes incubated in the absence of TLA extracts were used as non-stimulated controls. Lymphocyte proliferation was assessed using 3-(4,5-dimethylthiazol-2-yl)-5-(3-carboxymethoxyphenyl)-2-(4-sulfophenyl)-2H-tetrazolium, inner salt (MTS, 5 mg/mL, Promega) (MTS) assay. Absorbance was measured at 490 nm. The results were expressed as stimulation indices (SI), which were calculated as the ratio of OD_490_ values in vaccinated and control groups. The data represent three independent experiments.

### Measurement of cytokines

To characterize the cytokine level profile in response to immunization and prior to the challenge, we determined the concentrations of IFN-γ, IL-2, IL-4, IL-10, and IL-12p70 in splenocyte culture supernatants using Legend Max^TM^ ELISA Kits with Pre-coated Plates (Biolegend, San Diego, California, USA). Splenocytes obtained from immunized mice and their control counterparts at two weeks post final immunization were cultivated in 96-well plates. The splenocyte culture supernatants were collected at 24 h for the measurement of IL-2 and IL-4, at 72 h for the measurement of IL-10, and at 96 h for the measurement of IFN-γ and IL-12p70. All samples were tested in triplicate.

### Statistical analysis

All data were analyzed using the SPSS13.0 Data Editor (SPSS Inc., Chicago, Illinois, USA). Statistical differences among the four groups were determined by one-way analysis of variance (ANOVA) with the Tukey post hoc test. The differences in rates were analyzed by the student’s *t*-test. Data are expressed as the mean ± standard deviation (*SD*). The level of significance was defined as *p < 0.05 or **p < 0.01.

## Results

### *In vitro* expression of pVAX1-HSP40

**Figure 1.**
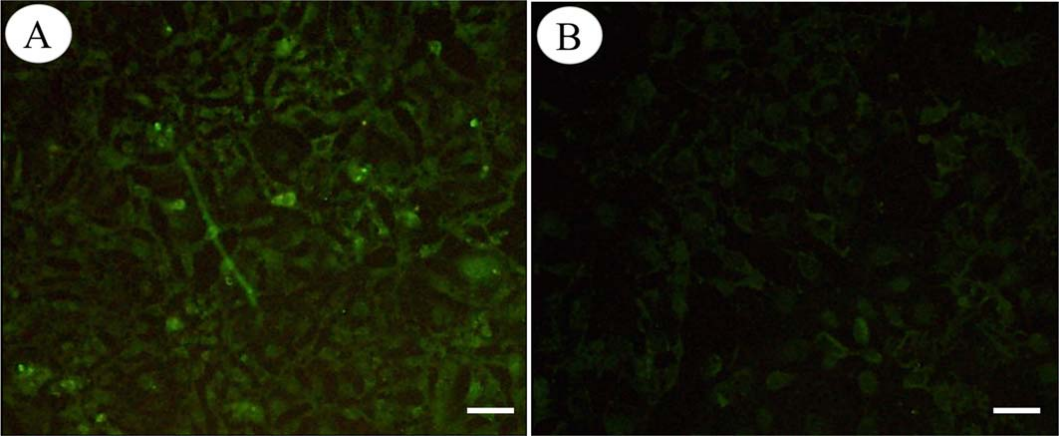
Immunofluorescence staining of HSP40 in HEK293 cells. HEK293 cells were transfected with pVAX1-HSP40 or pVAX1. At 72 h after transfection, the cells were fixed, permeabilized, and incubated with anti-*T. gondii* primary antibody. The cells were then incubated with fluorescein-conjugated secondary antibody and observed under a fluorescence microscope. The experiment was repeated three times, yielding similar results. The cells transfected with pVAX1-HSP40 showed the immunofluorescence of HSP40 proteins in the cytoplasm (A), whereas the cells transfected with the empty pVAX1 plasmid displayed little cellular immunofluorescence (B). Scale bar: 10 μm.

**Figure 2.**
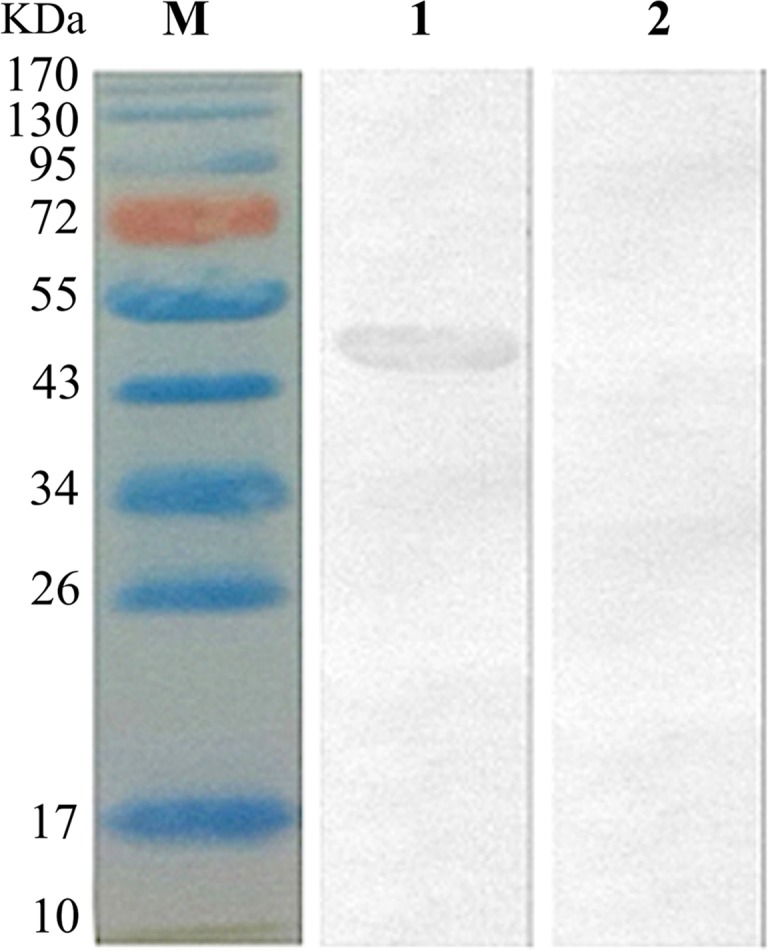
Western blotting of the expression of HSP40 protein encoded by the plasmid pVAX1-HSP40 *in vitro*. One band of approximately 46 kDa was detected in the pVAX1-HSP40-transfected cells (Lane 1), whereas no band was detected in protein extract of cells transfected with the pVAX1 control (Lane 2). M: protein marker.

The results of immunofluorescence staining showed immunofluorescence of HSP40 protein in the cytoplasm of pVAX1-HSP40-transfected HEK293 cells (Fig. 1A), whereas cells transfected with pVAX1 displayed little cellular immunofluorescence (Fig. 1B). Western blotting analysis showed a single band (~46 kDa) of the expected molecular size, indicating that pVAX1-HSP40 has been successfully expressed in eukaryotic HEK293 cells (Fig. 2). There was no protein band detected in the lysates of cells transfected with the empty pVAX1 vector.

### pVAX1-HSP40 protects mice against chronic infection

**Figure 3.**
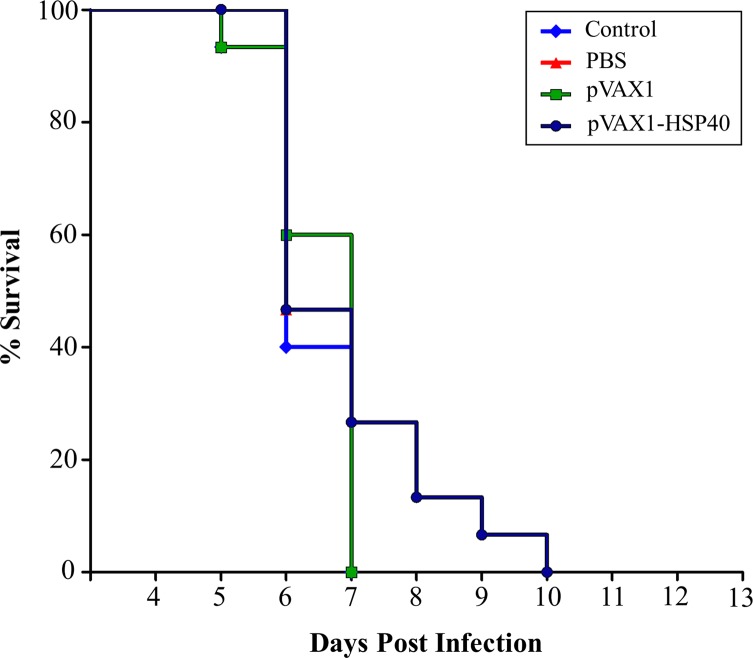
pVAX1-HSP40-based immunization failed to enhance the survival of acutely infected mice. Immunized and non-immunized mice were intraperitoneally challenged with 1 × 10^3^ tachyzoites of *T. gondii* RH strain at two weeks post the final immunization and their survival times were recorded daily until all mice died. No statistically significant difference was observed between control mouse groups (*p* > 0.05).

**Figure 4.**
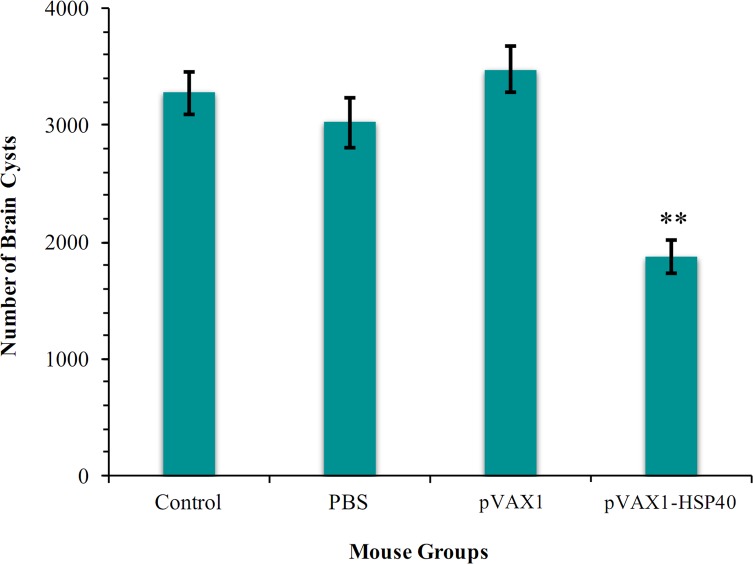
pVAX1-HSP40-based immunization protected against chronic infection. Number of brain cysts in mice challenged orally with 10 Pru cysts two weeks post the final immunization. The brain cysts in the pVAX1-HSP40-immunized mice were significantly lower compared with those in the control mouse groups (**p < 0.01). No significant difference was detected between the control mouse groups (*p* > 0.05).

After three booster immunizations using pVAX1-HSP40 with two-week intervals, Kunming mice were challenged with 10^3^ tachyzoites of *T. gondii* RH strain or 10 cysts of Pru strain in order to evaluate the protective efficacies against acute infection and chronic infection, respectively. The survival of immunized and non-immunized mice following challenge with *T. gondii* RH strain was monitored daily. As shown in Figure 3, no significant difference in the survival between pVAX1-HSP40-immunized mice (6.93 ± 1.28 d) and mice in the non-immunized groups (6.53 ± 0.64 d for pVAX1, 6.40 ± 0.63 d for PBS, and 6.33 ± 0.62 d for healthy control) was detected (*p* > 0.05). Interestingly, pVAX1-HSP40 immunization achieved a significant reduction in the parasite cyst burden in the brain of pVAX1-HSP40-immunized mice (1871.9 ± 142.3) compared with that of mice in the control groups immunized with pVAX1 (3479.2 ± 204.4), PBS (3024.4 ± 212.8), and healthy control mice (3275.0 ± 179.8), achieving 53.8%, 61.8%, and 57% reduction, respectively (*p* < 0.01; Fig. 4).

### Determination of T lymphocytes in spleen tissues

**Figure 5.**
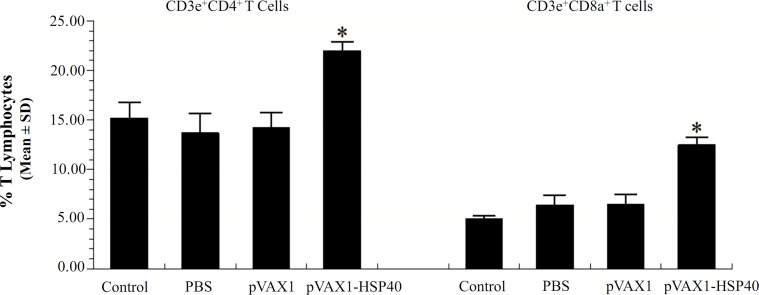
Flow Cytometry analysis of the proportion of T lymphocyte subclasses in mouse spleen. The CD3e^+^CD4^+^ T- and CD3e^+^CD8a^+^ T-lymphocytes in spleen of pVAX1-HSP40-immunized mice were increased significantly compared with that of mice in the control groups (*p* < 0.05). No significant difference was detected among the mice in the three control groups (*p* > 0.05).

The percentages of CD3e^+^CD4^+^ T and CD3e^+^CD8a^+^ T lymphocytes in spleen of immunized mice prior to challenge were determined using flow cytometry. We observed significant increases in the percentages of CD3e^+^CD4^+^ and CD3e^+^CD8a^+^ T lymphocytes in pVAX1-HSP40-immunized mice (22.0 ± 0.86 and 12.5 ± 0.78, respectively) compared with mice vaccinated with pVAX1 (14.2 ± 1.53 and 6.5 ± 0.98); with PBS (13.7 ± 1.96 and 6.4 ± 1.02) and in mice left non-immunized as healthy controls (15.2 ± 1.54 and 5.0 ± 0.33) (*p* < 0.05; Fig. 5).

### Measurement of specific antibodies in serum samples

**Figure 6.**
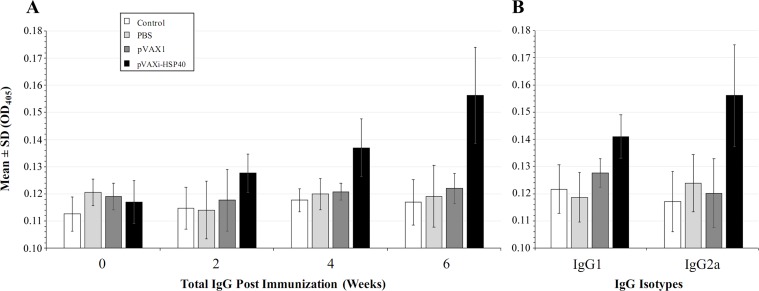
Levels of specific anti-rHSP40 antibodies in the serum of pVAX1-HSP40-immunized mice. (A) The optical densities (OD_405_) of total IgG prior to each of the three booster immunizations at 0, 2 and 4 weeks, and prior to the challenge at 6 week (i.e., two weeks after the third immunization) using ELISA. (B) Levels of IgG isotypes, IgG1 and IgG2a, two weeks after third immunization (i.e., 6 weeks after the primary immunization). There was no significant difference in the level of antibodies between immunized and control mice (*p* > 0.05).

As shown in Figure 6A, the levels of specific anti-*T. gondii* rHSP40 antibodies in the mouse sera examined prior to each of the three immunization doses, and prior to the challenge, were not significantly different between pVAX1-HSP40-immunized mice and mice from the three control groups (pVAX1, PBS and healthy control) (*p* > 0.05). The levels of IgG1 and IgG2a were determined two weeks after the third immunization. One way ANOVA and Tukey’s multiple comparisons test were used for statistical analysis of IgG1 and IgG2a data. The level of IgG2a was slightly increased in the pVAX1-HSP40-immunized mice compared with mice in all control groups, but the results was not statistically significant (*p* > 0.05; Fig. 6B).

### pVAX1-HSP40 immunization did not induce lymphocyte proliferation

**Figure 7.**
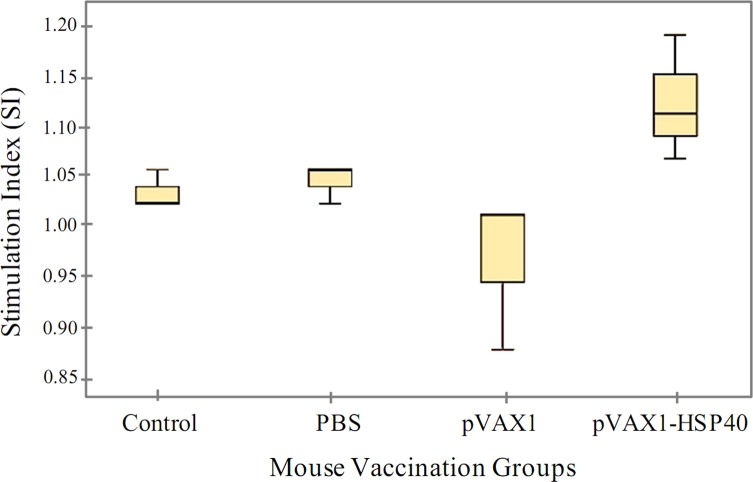
Lymphocyte proliferation assay. Splenocytes were obtained from immunized mice and the control groups prior to the challenge and cultivated in 96-well plates. Lymphocyte stimulation index was determined using the MTS assay. No significant difference was detected between pVAX1-HSP40-immunized group and control mice (*p* > 0.05).

The *in vitro* lymphocyte proliferation assay revealed that stimulation index of splenic lymphocytes from pVAX1-HSP40-immunized mice was not significantly different (*p* < 0.05) when compared with that of non-immunized controls, either in the presence of ConA or TLA (Fig. 7). This finding suggests a lack of induction of lymphocyte proliferation responses to TLA after vaccination with pVAX1-HSP40.

### Immunization did not influence the production of cytokines

**Figure 8.**
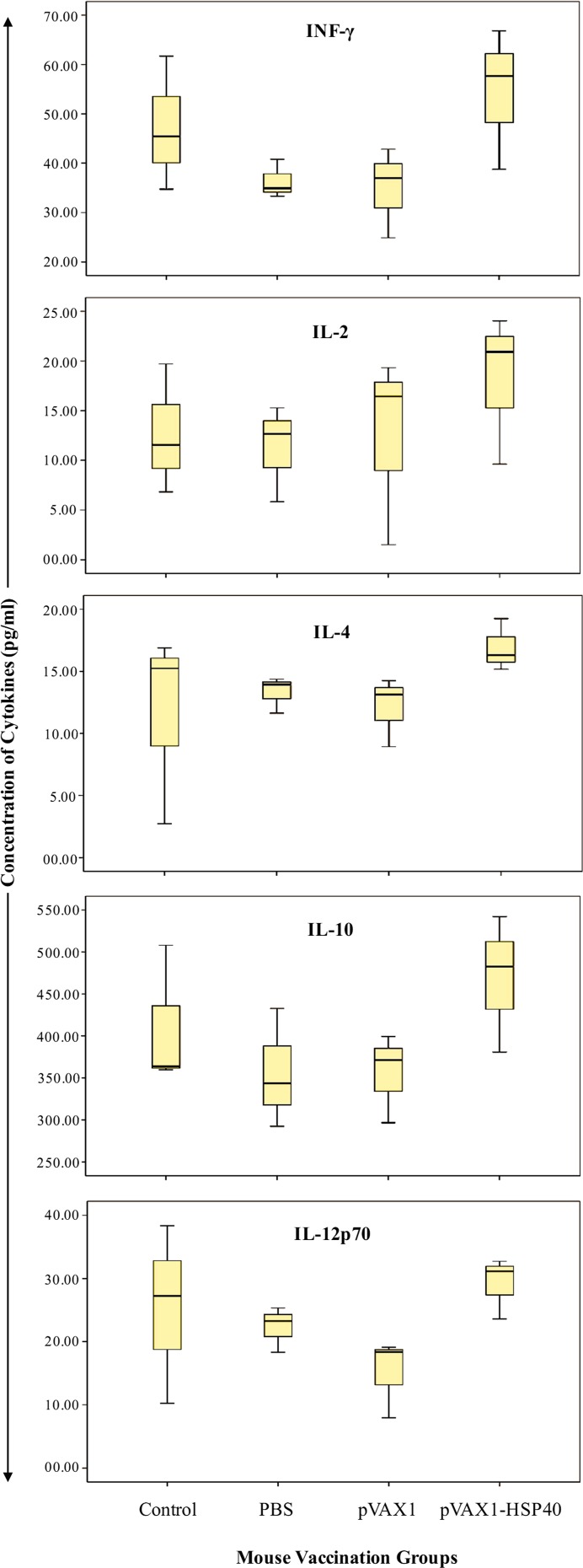
Levels of cytokines in splenocyte culture. Splenocytes were obtained from immunized and control mice prior to the challenge and cultivated in 96-well plates. The levels of IFN-γ, IL-2, IL-4, IL-10, and IL-12p70 cytokines in splenic culture supernatants were determined using ELISA. No significant difference was detected between pVAX1-HSP40-immunized mice and control mouse groups (*p* > 0.05).

The concentrations of five cytokines (IFN-γ, IL-2, IL-4, IL-10, and IL-12p70) in splenic culture supernatants (Fig. 8) were not significantly different between immunized and control mice (*p* > 0.05).

## Discussion

In the past few years, several attempts have been made to develop a preventative strategy to control *T. gondii* infection, such as using virulence-attenuated parasite strain and sub-unit vaccines [2, 17, 18, 35, 39]. Currently, leveraging the eukaryotic expression vector pVAX1 to develop DNA vaccine has been the focus of intense research because it can activate both innate and adaptive immune responses [41]. Interestingly, HSP40 has been associated with the virulence of a number of viruses and Apicomplexan protozoan parasites, and is consecutively expressed during the growth cycle of *T. gondii*, making it a good vaccine candidate [4, 21, 30]. For these reasons, the present study aimed to evaluate the protective efficacy of pVAX1 encoding HSP40 against *T. gondii* infection in mice.

The recombinant DNA plasmid pVAX1-HSP40 was successfully constructed and the expression of HSP40 in the transfected HEK293 cells was confirmed using an indirect immunofluorescent assay and Western blotting. This recombinant plasmid was used to immunize SPF-grade female Kunming mice for three sequential times with two-week intervals. pVAX1-HSP40-immunized mice developed fewer parasite cysts in their brain following oral challenge with 10 Pru cysts compared with non-immunized control mice. Specifically, pVAX1-HSP40 achieved 53.8%, 61.8% and 57% reduction in the parasite cyst burden, compared with cyst burden using vaccination with pVAX1, PBS, and healthy control, respectively. This result was higher than that obtained by DNA vaccine expressing *T. gondii* CDPK3 [40] and 39.08% by DNA plasmid (pVAX1-PF) encoding TgPF gene [13]. However, our result was comparable to the 57.8% reduction achieved by vaccination using multiple antigenic peptides encapsulated by chitosan microspheres [16].

In a striking contrast, the survival time of mice was not significantly different between mouse groups challenged with RH strain, indicating that sequential immunization with pVAX1-HSP40 was effective at preventing the chronic, but not acute infection with this parasite. Interestingly, the percentages of CD3e^+^CD4^+^ T and CD3e^+^CD8a^+^ T lymphocytes in the spleen of pVAX1-HSP40-immunized mice were significantly increased in comparison with control groups, which is consistent with previous studies [22, 38]. Both CD8^+^ and CD4^+^ T cells play an important role in resistance to *T. gondii* in mice [14, 26]; this was also indicated by the susceptibility of individuals with T cell deficiencies to toxoplasmosis [25]. Thus, the significant activation of the subclasses of T lymphocytes in spleen of immunized mice may be a contributing factor to the observed reduction in parasite cysts in the brain of immunized mice challenged with *T. gondii* Pru strain.

Infection with *T. gondii* can trigger strong innate and adaptive immune responses in the infected hosts [10, 28]. Th1-type cellular immune response during *T. gondii* infection plays an important role in protecting hosts from acute infection [11, 31]. Also, CD4^+^ cells and humoral antibody response contribute to controlling *T. gondii* during chronic infection by limiting development of the tissue cysts [19]. Therefore, specific anti-*T. gondii* antibodies including total IgG and its subclasses (IgG1 and IgG2a) were examined. Similar to other *T. gondii* virulence factors, such as rhoptry protein 18 [38], immunization with pVAX1-HSP40 was able to slightly increase the production of specific antibodies (*p* > 0.05) and only after the third booster immunization, indicating that the strategy of using multiple sequential doses was necessary to enhance the humoral immune response in the immunized mice. A mixed Th1/Th2 immune response, slightly skewed towards Th1-type response as indicated by a slightly higher lgG2a/IgG1 ratio (*p* > 0.05), was detected in pVAX1-HSP40-immunized mice (Fig. 6B). This result suggests that vaccination using pVAX1-HSP40 can elicit a cell-mediated immune response, which was anticipated from a DNA vaccine. However, this finding was not supported by the results of the lymphocyte proliferation assay or the concentrations of cytokines (IFN-γ, IL-2, IL-4, IL-10, and IL-12p70), which were not significantly different between immunized and non-immunized mice.

In conclusion, sequential immunizations with pVAX1-HSP40 induced cellular immune responses and reduced the number of brain cysts in mice challenged orally with Pru strain. However, immunization had no influence on lymphocyte proliferation or the levels of cytokines. These data suggest that *T. gondii* HSP40 may be a good DNA vaccine candidate, which can be used together with other parasite proteins to formulate a cocktail vaccine against chronic *T. gondii* infection.
